# Supplementary material for: The Relationship between Angiogenic Factors and Energy Metabolism in Preeclampsia
Source: Nutrients. 2022 May 23;14(10):2172. doi: 10.3390/nu14102172 (PMC9145768; doi:10.3390/nu14102172)
Supplement: Supplementary file 1 [file nutrients-14-02172-s001.zip › nutrients-1715636-supplementary.pdf]

Table S1. Maternal, obstetric and perinatal results: EOPE vs. LOPE.

**MATERNAL RESULTS**	**EOPE** *n* = 11	**LOPE** *n* = 15	*p* **value**
Maternal age (years)	33.4 ± 7.1	35.4 ± 2.7	0.380
Ethnic Group:			0.565
• Caucasian	7 [63.6%]	7 [58.3%]	
• Hispanic	4 [36.4%]	4 [33.3%]	
• Asian	0	1 [8.3%]	
Pregravid body mass index (kg/m2)	22.2 ± 3.2	24.8 ± 3.8	0.125
Body mass index classification:			
• Underweight (< 18.4)	1 [12.5%]	0	0.276
• Normal weight (18.5-24.9)	5 [62.5%]	7 [58.3%]	0.052
• Overweight (25.0-29.9)	2 [25.0%]	4 [33.3%]	0.166
• Obesity (> 30.0)	0	1 [8.3%]	0.276
Gestational weight gain (kg)	9.0 (4.0)	14.0 (10.00)	0.017*

**OBSTETRIC RESULTS**			
Gestational age at study (weeks)	34.0 (3.0)	37.0 (1.0)	0.000211*
Parity:			
• Primigravid	6 [54.5%]	7 [46.7%]	0.580
• Multiparous	2 [18.2%]	3 [20.0%]	0.640
• Previous C-section	2 [18.2%]	3 [20.0%]	0.585
• Previous miscarriage	1 [9.0%]	6 [40.0%]	0.230
Mode of pregnancy:			0.531
• Spontaneous	8 [72.8%]	10 [76.9%]	
• ART (IUI)	0	0	
• ART (IVF)	2 [18.2%]	3 [23.1%]	
Twin pregnancy	2 [18.2%]	4 [26.7%]	0.003*
Obstetric reason for caesarean section:			
A.- Fetal:			0.227
• Breech or transverse fetal position	1 [9.1%]	1 [7.1%]	
• Suspicion of fetal macrosomia	0	0	
• Intrapartum fetal distress	1 [9.1%]	1 [7.1%]	
B.- Maternal:			0.366
• Preeclampsia	9 [81.8%]	8 [57.1%]	
• Iterative C-section or Previous C-section + Bishop test ≤ 6	0	0	
• Twin pregnancy + Bishop test ≤ 6	0	0	
• Labor dystocia	0	3 [21.4%]	
• Elective	0	0	
• Fracture of femur head	0	0	
• Myopia magna	0	1 [7.1%]	

**PERINATAL RESULTS**			
EFW (g)	1307.9 ± 500.1	2322.2 ± 556.3	0.000068*
Centile EFW	9.5 ± 16.9	54.1 ± 40.8	0.001*
Fetal growth restriction:			
• SGA	3 [27.3%]	0	0.124
• IUGR	8 [72.7%]	3 [20.0%]	0.354
Neonatal birth weight (g)	1334.4 ± 533.2	2582.5 ± 728.1	0.000045*
Neonatal birth centile weight	1.7 ± 2.2	52.2 ± 37.8	0.000004*
Neonatal sex:			0.075
• Male	2 [15.4%]	9 [50.0%]	
• Female	11 [84.6%]	9 [50.0%]	

Umbilical artery pH at birth	7.29 ± 0.06	7.28 ± 0.06	0.675
Placental weight	295.7 ± 107.9	472.5 ± 135.9	0.001*

Key: EOPE: early-onset preeclampsia, LOPE: late-onset preeclampsia, C-section: caesarean section, ART: assisted reproductive technology, IUI: intrauterine insemination, IVF: in vitro fertilization, EFW: estimated fetal weight, SGA: small for gestational age, IUGR: intrauterine growth restriction. The results of qualitative variables are represented as absolute values and percentages, n [%]. The results of quantitative variables are expressed as mean ± standard deviation, or as the median and interquartile range (in brackets) according to the distribution of the variable. * The difference was significant compared to both groups ($p < 0.05$).
